# Functional Assessment for Acute Stroke Trials: Properties, Analysis, and Application

**DOI:** 10.3389/fneur.2018.00191

**Published:** 2018-03-26

**Authors:** Martin Taylor-Rowan, Alastair Wilson, Jesse Dawson, Terence J. Quinn

**Affiliations:** Institute of Cardiovascular and Medical Sciences, University of Glasgow, Glasgow, United Kingdom

**Keywords:** stroke, outcome, disability, modified Rankin scale, Barthel index, NIHSS

## Abstract

A measure of treatment effect is needed to assess the utility of any novel intervention in acute stroke. For a potentially disabling condition such as stroke, outcomes of interest should include some measure of functional recovery. There are many functional outcome assessments that can be used after stroke. In this narrative review, we discuss exemplars of assessments that describe impairment, activity, participation, and quality of life. We will consider the psychometric properties of assessment scales in the context of stroke trials, focusing on validity, reliability, responsiveness, and feasibility. We will consider approaches to the analysis of functional outcome measures, including novel statistical approaches. Finally, we will discuss how advances in audiovisual and information technology could further improve outcome assessment in trials.

## Introduction

Clinical trials are designed to assess the effect of a novel intervention versus a comparator. An archetypal stroke trial may, for example, assess the effect of endovascular treatment against a control of “usual care.” The PICO framework can be used to describe any clinical trial in terms of Population, Intervention, Control, and Outcome. While it is typically the intervention that attracts attention and represents the exciting new chapter in stroke care, we should not forget about the other components of a trial. In particular, outcome assessment in stroke trials is critical and the approach to outcome assessment can be the difference between a positive and neutral trial.

The outcome of any trial should provide some quantifiable measure of the effect of the treatment. Historically, endpoints such as mortality or event recurrence have been used in stroke trials. While useful, particularly for trials of primary and secondary prevention, these “hard clinical endpoints” do not capture the full extent of outcomes for a disabling condition such as stroke. Therefore, assessment of patients’ functional ability has been adopted and is now mandated by regulatory authorities for certain stroke trials. Multiple measures of post-stroke functional ability have been developed and many have been used in stroke trials.

In this review, commissioned as part of the themed series on hyper-acute stroke trials, we discuss commonly used functional outcome measures in these trials. We briefly describe their historical purpose before evaluating each in relation to core psychometric properties (see Table 1). We then discuss analytical approaches that can be used to assess stroke functional outcomes. Finally, we consider how training, structured assessment, and advances in technology may enhance stroke outcome assessment.

**Table 1:** Core psychometric properties and how we evaluate them.

Psychometric property	Domain and definition	Statistical analysis
Validity: the degree to which a tool measures what it purports to measure		Established *via* correlation e.g., Pearson’s “r” or Spearman’s “rho”:1.0 is a perfect correlation; 0.0 suggests no correlation
Concurrent validity	The extent to which a tool results correspond to other measures associated with the outcome of interests (i.e., functional disability)	
Construct validity	A tools association with other tools that measure the same, or a similar construct	

Predictive validity	Ability of the tool to predict future events	Established *via* odds ratios (OR) e.g., OR:2.00 suggests two times greater odds of an outcome occurring when variable *x* is present than when not

Reliability: refers to a tools consistency in scoring over multiple assessments		Established *via* kappa (k) or interclass correlation coefficient (ICC) values.Both values range between 0 and 1 with values closer to 1 indicating greater reliability
Inter-rater reliability	Consistency of scoring across different assessors	
Intra-rater reliability	Consistency of scoring within the same assessor	

Internal consistency	Agreement between items within a multiitem scale	Established using Cronbach’s alpha

Responsiveness: the tools ability to detect meaningful change over time		Determined based upon a tool’s sensitivity to improvement or decline with repeated testing
Feasibility: the practicality or reasonableness with which a tool can be used. Can incorporate measures of acceptability to rater and patient		Ratio or percentage of patients with which the assessment could be performed

## A Framework for Considering Outcome Assessment

**Figure 1:**
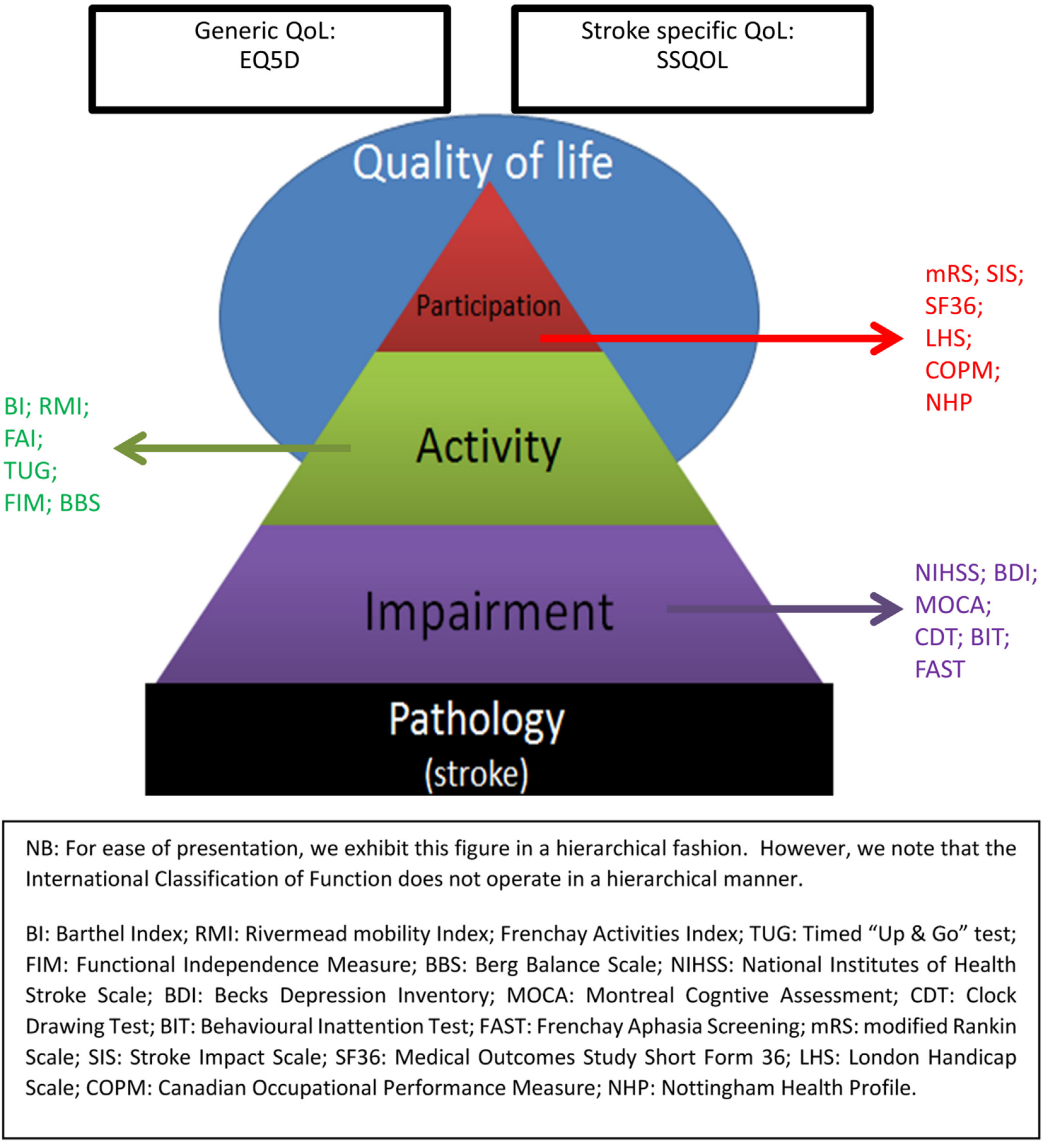
World Health Organization International Classification of Function (WHO-ICF) Assessment Scales.

There are numerous potential outcome assessments for stroke research (1). For example, even in a relatively niche area such as post-stroke psychological assessment, recent reviews have found more outcomes than trials (2). With such a range of potential functional assessment tools, it is useful to have a framework for considering the application of these tests. Our goal is functional outcome assessment, but “functional outcome” is a broad term that encompasses many constructs. A potentially useful way to categorize functional outcomes is to consider the World Health Organization International Classification of Function (WHO-ICF). The WHO-ICF describes function in terms of impairment, activity (formerly disability), participation (formerly handicap) (3), and we could add a fourth level of quality of life (QOL). Stroke assessment scales are available to describe functional outcome at each level of the WHO-ICF (Figure 1).

**Figure 2:**
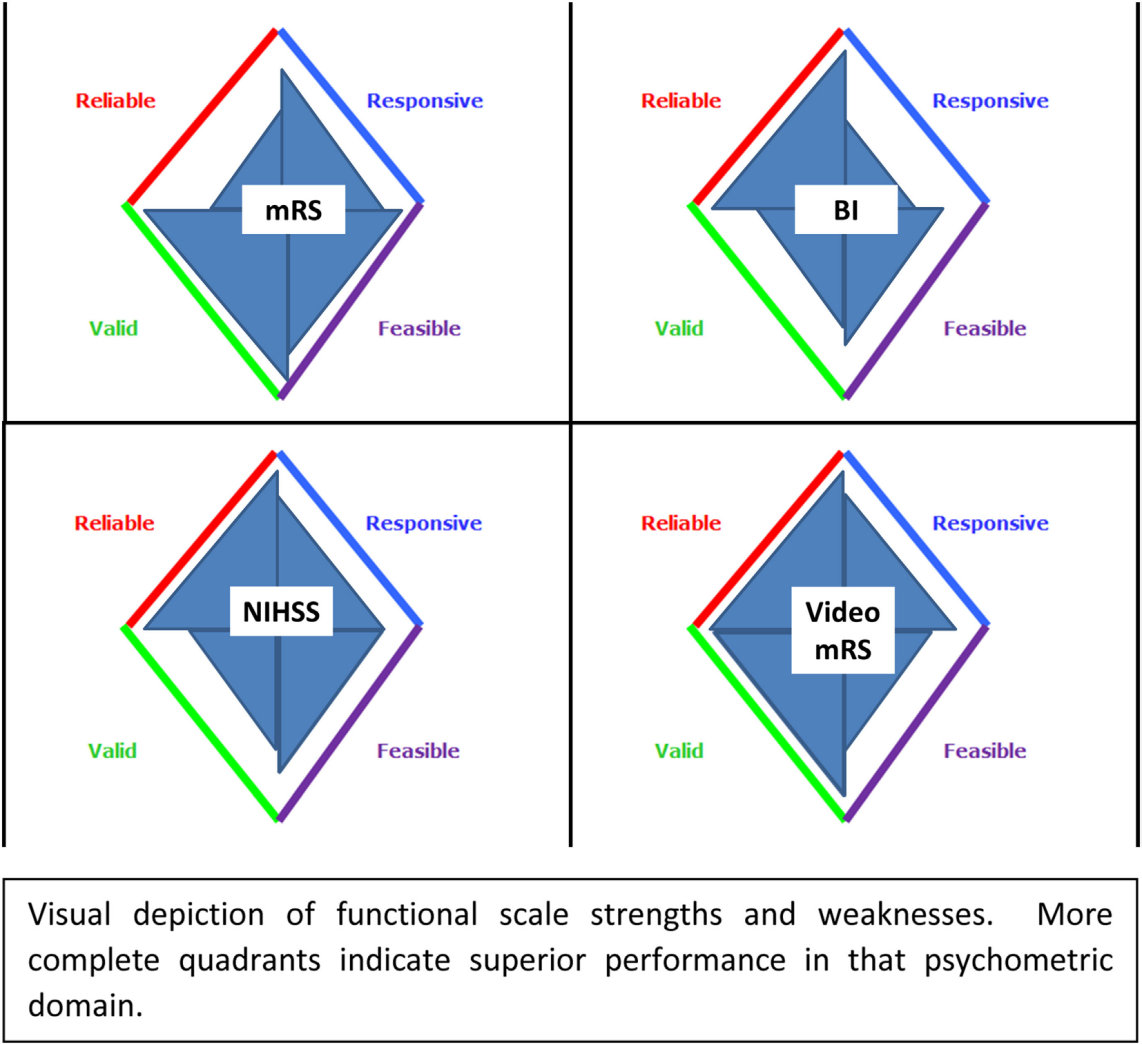
Functional scale psychometric properties.

Even within each level of WHO-ICF, there can be many potential assessments to choose from. The science of psychometrics (sometimes called clinimetrics in the applied clinical context) describes properties of assessment scales. The classical properties that are important for a clinical assessment tool are validity, reliability, responsiveness to change, and feasibility/acceptability (4). Depending on the clinical context and population to be studied, some psychometric properties may be more important than others (Figure 2).

We will consider three outcome assessment scales that have been frequently used in acute stroke trials, each one has been chosen as an exemplar of a certain level of the original WHO-ICF framework. For each assessment scale, we will describe all four of the classical psychometric properties, using each scale to major on a particular aspect relevant to that scale.

Research into the properties of stroke scales is an evolving field. In this review, we highlight many of the seminal papers that have influenced our understanding of stroke clinimetrics. We recognize that in some instances authors may have used statistical approaches that are not reflective of current best practice. For example, statistical analyses based on parametric assumptions have often been applied to stroke scales that are ordinal or nominal in structure. While some argue that parametric statistics are inappropriate for evaluating stroke scales, it would be wrong to ignore all the available research that has used this approach. We also note that variations in language and translations can potentially affect scale properties. Our discussion will predominantly focus on the original (English language) versions of these tools.

## Impairment: National Institutes of Health Stroke Scale

### History and Purpose

The NIHSS was specifically designed for assessment of interventions in clinical trials. Of key intent was that the tool should be employed easily and quickly at the patient bedside to enable practicality of use (5). Rather than measuring function specifically, the NIHSS operates a 15-item ordinal, non-linear, neurological impairment scale covering consciousness, ocular movement, vision, coordination, speech and language, sensory function, upper and lower limb strength, facial muscle function, and hemi-neglect (6). Initial piloting took place in a controlled acute stroke trial assessing the effects of naloxone; it is now commonly used in acute-clinical stroke practice (see Table S1 in Supplementary Material).

### Validity

The NIHSS’ attention to specific neurological deficits engenders a high-concurrent validity (0.4–0.8) based upon association with infarct size (5, 7). Information on construct validity is lacking. The tool is well suited to early stroke severity assessment and baseline scores have strong predictive validity with outcome at 7 days and 3 months (8). Specifically, patients with a baseline score of <5 are almost always (80%) discharged home; scores of 6–13 often need inpatient rehabilitation; and scores of 14+ are strongly associated with need for longer-term care.

There are however questions as to how well the tool determines “real world” functional impact. For example, a lesion that results in a hemianopia and a score of “1” on the NIHSS would typically be categorized as a “good” outcome (9). Yet, if such impairment precludes driving, consequences upon employment, independence and mood could be substantial. Additionally, the focus of the tool is weighted toward limb and speech impairments with reduced attention to cranial nerve-related lesions (10) and appears to have reduced validity when lesions present in the non-dominant hemisphere (11).

### Reliability

The scale has exhibited excellent inter (ICC = 0.95) and intra-observer reliability (ICC = 0.93). The high inter-rater reliability is observed in both neurologically trained and non-neurologically trained raters alike (11).

### Responsiveness

The responsiveness of the tool has compared favorably to both the BI and mRS in detecting a treatment effect (5, 9).

### Feasibility

The NIHSS is optimally generated using a formal observational patient assessment. Recognizing that unwell patients may be unable to participate in all aspects of testing, there is scoring guidance for incomplete test items. On average, NIHSS assessment takes around 5 min to complete. For retrospective assessment in audit or research, the NIHSS can be derived using medical records (12); this is not true for more complex assessments such as mRS (13).

### Summary

The NIHSS has favorable properties, although as an impairment-based scale it is not a good measure of the broader disability that can result from a stroke. The NIHSS is perhaps best used as case-mix adjuster or early outcome assessment measure for hyper-acute trials.

## Activity: Barthel Index

### History and Purpose

Designed to measure independence, the Barthel index (BI) was originally used to assist patient discharge and long-term care planning in non-stroke settings (11). The BI operates according to a 10-item scale in which patients are judged upon degree of assistance required when carrying out a range of basic activities of daily living (ADL) (see Table S1 in Supplementary Material). The assessment is delivered through an established and validated questionnaire comprising a total score of 100 for the 10 items of the scale. The patient’s answers on each item are scored based upon actual ability (preferably observed by the assessor). The usual scoring for each item is 0 points for “no ability” to do the item independently, 5 points for “moderate help” with the item, and 10 points for being able to manage the item independently. The BI has emerged as the second most popular tool for assessment of post-stroke outcome in clinical stroke trials (1).

### Validity

Concurrent validity, appropriated *via* correlation with infarct size, extent of motor loss and nursing-time requirements, appears to be moderate (*r*^2^ = 0.3–0.5) (7, 14, 15). Construct validity is favorable when compared with other measures of activity (16), while predictive validity of the BI has been established on basis of low BI scores correlating with future disability, longer time to recovery, and heightened care needs (17). It is important to note, however, that the predictive validity of the BI can be suboptimal if it is conducted too early (within 5 days post-stroke) (18), and validity of the tool may be compromised by self-report measurement, particularly when cognitive impairment is present (19). Validity of the tool when used in the hyper-acute stroke period is also questionable as monitoring equipment, physical illness, and restrictions on mobility may all compromise the true score.

### Reliability

The inter and intra-reliability of the tool is judged to be moderate (*k* = 0.41–0.6) to high (*k* = 0.81–1.00) (20, 21). However, the studies from which this evidence pertains are limited in sample size and heterogeneous in both methodology and assessment quality (18). Of additional note, reliability seems to vary across specific items of the scale and is greatest at higher BI scores (22).

### Responsiveness

Responsiveness to change has been described as a strength of the BI over other stroke functional assessment tools (23–25). The overall responsiveness of the tool is reasonable within a certain range of disability; however, it also appears vulnerable to floor and ceiling effects, which are largely attributable to the scales’ assessment of basic ADLs only (11). Specifically, the tool is often insensitive to changes in patients whose general mobility and physical function is impaired, but who improve in other aspects—for example, cognitively (floor effect); or where there are limitations in extended ADL’s—for example, due to cognitive impairment (ceiling effect).

### Feasibility

The simple structure of the BI allows for direct assessment, proxy-based assessment, telephone assessment, and postal questionnaire. Where possible the information should be based on direct observation of the tasks. BI is relatively quick to perform, but for large-scale audit and research shorter versions have been developed. Recent efforts to enhance feasibility include a short-form version of the BI which includes three items: bladder control, mobility, and transfers (26). This version of the tool has been validated *via* systematic review of short-form BIs, and while validity is reduced by comparison to the full scale, it is no worse than longer versions containing four and five items (27).

### Summary

Although still a popular outcome measure, the BI has properties that limit its utility as primary endpoint in an acute stroke trial. In particular, for those trials where moderate-to-severe disability is not expected, the usefulness of BI is limited by an emphasis on basic ADLs and physical constructs. The BI is perhaps best used to assess case-mix and early outcomes in stroke rehabilitation settings.

## Participation: Modified Rankin Scale

### History and Purpose

Adapted from the original 1957 Rankin scale (28), which was designed to assess patient outcomes in one of the first stroke units, the modified Rankin scale (mRs) was the first functional outcome assessment used in a stroke trial. The mRS is the most commonly used functional assessment measure and is recommend by professional societies and regulatory bodies^1^ for outcomes assessment in stroke trials. The mRS adopts a 7-point hierarchical, ordinal scale to measure functional independence (see Table S1 in Supplementary Material).

There has been some debate as to the nature of mRS scoring. We have classified as a measure of participation for the purpose of this review, as the scale offers a broad focus potentially going beyond the basic and extended ADL measures of an activity scale. Other scales are available that are more clearly aligned with the concept of participation but these tools are rarely used in stroke trials (29) whereas mRS is a common outcome assessment that at least serves as a proxy of participation.

### Validity

Analysis of clinical properties suggests concurrent validity based on correlation coefficients with infarct volume is of 0.4–0.5 (30)—comparable to the BI (14, 15, 31). Assessment of construct validity suggests that the mRS has excellent agreement with other stroke functional scales (32), while predictive validity is demonstrated by the association of short to medium term mRS with longer-term post-stroke care needs (17). The validity of the scale can however be affected when a proxy is used to generate a score, or when applied in the acute setting during when the patient has not yet had the chance to resume normal activities. When used in a retrospective fashion to determine the pre-stroke functional state, the mRS validity can be diminished, demonstrating moderate-concurrent validity (ρ > 0.4) when compared with other variables associated with function (33).

### Reliability

Reliability, particularly inter-observer variability, has been identified as the main drawback of the scale. This is a consequence of the simplicity of the tool and its use of a 7-point scale, which is both shorter than many other assessments and less categorical in descriptors at each point, thus requiring greater interpretation from assessors (34). Meta-analysis suggests an inter-rater reliability of κ = 0.62; however, in multicenter trials this maybe be as low as κ = 0.25 (35). This can be further compromised when telephone assessments are utilized to conduct the assessment (36). Statistical noise generated by the poor inter-rater reliability of the mRS increases vulnerability to type-2 errors, meaning that clinically significant treatment effects can be missed. Some of these issues can however be potentially alleviated *via* structured interview, training and central adjudication, all of which we discuss below (37–39).

### Responsiveness

The mRS responsiveness to change has received comparatively less attention than the scales’ other properties. With a limited number of possible scores, the mRS may have inferior responsiveness to change compared with other measures of post-stroke function, although any change seen in the mRS is likely to be clinically meaningful. In a non-random sample of stroke rehab patients, the BI has demonstrated favorable responsiveness to change over the mRS (*p* = 0.002) (23). Further issues with regard to the responsiveness of the mRS over particularly short time-frames (i.e., admission to discharge) have also been highlighted (23).

### Feasibility

The traditional method for mRS assessment is an unstructured direct to patient interview. These interviews are usually short, but the open nature of mRS questions can lead to longer interviews while issues are explored to the satisfaction of the assessor. Where a patient is unable to fully participate in an interview, a proxy can be used (40). The use of structured interviews may improve reliability, although this has not been consistently proven, and short structured mRS assessments have been used in some trials (41).

### Summary

Overall, the mRS offers a brief yet broad ranging assessment of function. This comes at the price of reliability issues and potentially reduced responsiveness to subtle improvement or deterioration. As a global measure of functional recovery that captures clinically meaningful change, mRS is perhaps best suited as endpoint in large trials of potential stroke treatments.

## Quality of Life

Moving beyond the WHO-ICF construct of participation, one can consider a further level of potential outcome assessment as QOL. Again, there are various QOL tools available; for clinical purposes we usually consider health-related assessment scales (HR-QOL) and these can be generic (e.g., the various iterations of the Euro-QOL) or stroke specific (e.g., the Stroke Impact Scale). QOL assessments have particular utility as they can be used to inform health economic analyses (42). The use of HR-QOL assessments is increasing, in part driven by the recognition of the value of patient reported outcome measures. At time of writing no positive stroke trial has used an HR-QOL as primary outcome but this may soon change. In the longer-term post-stroke, QOL will be a product of many factors many of which may be unrelated to the stroke. There is a tension between having a tool that allows comprehensive assessment and having a tool that does not require a lengthy and burdensome interview. In this regard, recent attempts to create shorter HR-QOL forms that retain the most discriminating questions are welcome (43).

## Statistical Analysis of Functional Endpoints

The statistical approach to analysis of functional outcomes can have implications for sample size, validity and ultimately the success of the trial. In this section, we will mostly discuss the mRS but many of the themes regarding analysis will equally apply to other assessment scales.

### Composite Endpoints

So far we have considered functional assessment scales in isolation. However, as the scales assess differing constructs there could be advantage in combining endpoints. Indeed in the seminal NINDS trial of tPA, scores on NIHSS, BI, mRS, and Glasgow Outcome Scale were assessed in aggregate. The use of composites may have particular utility where outcomes individually may be uncommon. Using a modeling approach, the utility of a composite outcomes to improve power in a trial in minor stroke and acute ischemic stroke have been described (44). The main limitation of composites is in the interpretation and there can be problems, if, for example, a patient has a favorable outcome on one component of the composite and an unfavorable outcome on another. Also, if measures are not independent of one another, error measurement can be exacerbated and there can be a temptation to adopt this approach *post hoc* because individual measures are non-significant (45).

### Cut Points and Shift

The mRS offers ordinal, hierarchical data, and historically the most commonly applied approach to analyses was to dichotomize scales at a set cutoff point, thus distinguishing those who achieve a “good outcome” from those who do not. Although there have been attempts to define an optimal cut point for the differing outcome assessments (9, 46), this approach is slightly misleading as the optimal cut point will vary with the population studied and the anticipation of functional recovery. So, for example, in studies of decompressive hemicraniectomy, a “good” outcome could be defined as less than or equal to mRS 3, while in a trial of tPA for minor stroke one would define a good outcome at a much narrower range, for example, mRS 0–1. What is clear from all the studies of dichotomized cut points is that the choice of scale and cut point will dictate the required sample size to demonstrate a treatment effect.

Dichotomization offers relatively simple comparative analyses, but this reductionist good versus bad outcome approach can miss important treatment effects and will be insensitive to partial, but meaningful improvements in functioning, such as an increase from a score of 5 to 3 in the mRS (i.e., an improvement from bed-ridden to independent mobility, a change which most would accept as clinically important). Indeed, adoption of a dichotomization approach has been implicated in false-neutral findings of stroke trials with examples of trials where dichotomized outcomes potentially missed a treatment effect that was observed using other approaches and examples of where dichotomized outcomes may have provided missed potentially harmful interventions (47).

It is possible to apply a prognosis adjusted endpoint method to analyses, whereby “good” outcomes are defined by achieving a standard dichotomized “good outcome” or by extent of improvement across the scale (e.g., an improvement in score of *n* points on NIHSS). This moderates some of the statistical limitations inherent to dichotomization as it allows more patients’ data to contribute to the results (9). Again, however, there is some uncertainty as to how such “good outcomes” should be defined regarding the extent of change in score. Research into the NIHSS suggests the most discriminating prognosis adjusted endpoint appears to be a score of 1 or less overall, or a change in score of 11 points (9).

The alternative approach to segregating data and analyzing on the basis of dichotomized “good outcomes” is to evaluate more of the scale. Trichotomized endpoint analyses have been described but have been superseded by techniques that allow assessment of the entire ordinal scale range *via* a shift analysis. Such approaches that exploit the full distribution of outcomes include the proportional odds model and the Cochran–Mantel–Haenszel test. Shift analysis has been suggested to improve the overall power that can be generated compared with a dichotomized mRS (47). This seems to be particularly true when treatment effects are small, but uniform over all respective ranges of stroke severity (although dichotomization can be mildly more powerful than shift analysis when treatment effects are substantial in certain circumstances) (47). The potential utility of the shift analysis over dichotomization has been demonstrated empirically in recent trials. For example, the INTERACT-2 study of blood pressure reduction in intracerebral hemorrhage was neutral on a primary endpoint of dichotomized mRS, but demonstrated a treatment effect on prespecified secondary shift analyses (48).

A shift approach to mRS assessment is gaining traction in stroke research but we must be mindful of potential limitations in this approach. The main issue with this method is that there are implicit assumptions inherent to shift analysis that may not hold when applied to ordinal scales such as the mRS. For example, the Cochran–Mantel–Haenszel method of analysis assumes that treatment effects are uniform over the full range of the mRS scale; that is, that the treatment effects will be the same for those scoring mRS 0–1 as it is for those scoring mRS 2–3 (49, 50). Moreover, shift analysis is typically considered superior to dichotomized analysis when the error is uniform across the scale. However, inter-rater reliability and misclassification errors are often most problematic in the mid-range (mRS 2–4) of the mRS scale, meaning that errors are typically not evenly distributed (51). When error rates are high and non-uniform, shift analysis may reduce power by comparison to dichotomization (52). Due to these issues, some authors (45, 52, 53) advise against employing shift analysis to ordinal scales such as the mRS, particularly in early-phase trials with small patient samples.

### Utility Weighting

A novel approach to assessment that has been used in contemporary endovascular studies is to apply weighting to outcomes. In utility weighting, it is recognized that certain health outcomes and transitions between outcomes will be more desirable than others. A utility weighted mRS has been developed that incorporates patient and societal valuations of each potential mRS outcome (54). The weighting can be performed by mapping EQ-5D population data onto mRS or using disability weighting. Potential advantages of utility weighted mRS have been demonstrated using secondary analysis of existing trial data (54) but the real proof of the value of the utility weighted approach comes from the recent DAWN trial (55). As the greatest utility values are assigned to transitions from high disability states to lower, then the utility-based approach may have particular value in treatments with the potential to prevent disability such as large artery thrombectomy. It is however important to note that in adopting this approach the higher ends of the mRS scale are filtered out, which may produce a non-Gaussian distribution. Hence, this method can result in the inappropriate application of standard statistical tests.

## Future Directions in Stroke Outcome Assessment

Even with an appropriate outcome measure and statistical analysis plan, demonstrating a treatment effect of a stroke intervention is not easy. Developments in audiovisual and information technology and best practice guidance in outcome assessment is helping to raise standards and improve the application of stroke outcome assessments.

### Training

Although the assessment scales discussed are theoretically objective, there is always a degree of subjective interpretation. To ensure standardization of assessment, scoring rules and training materials have been developed. Direct training from an experienced assessor is not possible at scale across the many international sites that may participate in a stroke RCT. Training manuals and use of audiovisual materials is one potential solution. For example, mass training in NIHSS using video-recorded patient assessments has proven feasible and popular (56). The format has evolved with changes in available technology from videotape recordings (57), to DVD and now interactive online materials (58). Completion of NIHSS training has been shown to improve scoring and a certificate of completion of NIHSS training is now mandatory for many studies where NIHSS is an outcome measure. Similar resources are available for mRS (59) and BI and also seem to improve application of these scales (60). The mRS training is similar to NIHSS with teaching cases, tutorials, and a certification exam.^2^ BI training is a descriptive tutorial rather than video-based patient assessment (see text footnote 2). Although the use of these mRS and BI training materials seems intuitively attractive, there have been no suitably large trials that have demonstrated improvements in scoring with training. Nonetheless, it seems unlikely that training would worsen performance in assessment and so we would advocate continued use of such resources.

### Structured Assessments

The mRS and to a lesser extent the BI are based on an interview with the patient. To ensure interviews are focused and have consistency of content, a series of structured mRS’ have been proposed. These can be structured, anchoring questions with guidance on interpretation or more formal questionnaires with a series of yes/no responses. Advocates of the structured approach report less time spent on interview and improved reliability. However, proponents of a less-structured interview note the benefits of a flexible approach. A structured interview can result in the discarding of essential information when contemplating a patient’s functional ability, particularly concerning usual activities such as work or hobbies. Moreover, if a patient’s answers do not “fit nicely” with a given item in the questionnaire, the rigid structured nature of the interview can be a hindrance rather than a benefit. A systematic review and meta-analysis that pooled all available data did not find benefits of structured interview over standard face-to-face interview, albeit some of the structured interviews used in contemporary trials were not available at the time of the review (61).

### Centralized Adjudication

Expert group adjudication of outcome measures such as neuro-imaging or electrocardiographs (ECG) has been routinely used in multicenter clinical trials as a method of reducing inter-observer variability and maintaining quality control. In contrast, traditionally functional outcomes were only assessed at participating sites, but the landscape is changing.

As mRS and to a lesser extent BI can be scored based on an interview, both have the potential for telephone administration. The properties of telephone mRS and BI are less well described than direct assessment and there may be some systematic differences in scoring. However, telephone assessment is attractive for a large multisite study, as it saves time, reduces patient/assessor travel and reduces test burden. In terms of centralized assessment, if telephone interviews are coordinated from a single center there can be more consistency of assessment and easier quality control. Telephone assessments can be audio recorded for off-line assessment by an adjudication panel. These processes were used for a subset of assessments in a recent thrombectomy trial (62).

Audio recording only gives a partial assessment and with the increasing availability of affordable portal video-recording equipment and high-speed data transfer there is increasing potential for audiovisual recording of stroke assessment. Such video assessment allows for remote centralized adjudication of any functional outcome assessment.

Centralized adjudication of the mRS has been employed in international trials with recruitment from a diverse range of countries from Vietnam to Kazakhstan and both North and South America (37). In this particular video-based platform, typically, the centralized adjudication of Rankin scoring employs a panel of 2 or more raters from a pool of expert assessors to score the mRS of the patient. A final score is assigned by a committee based on consensus agreement.

While video-based centralized adjudication necessitates an additional initial cost to the trialists, the availability of low-cost video-recording equipment and high-speed data transfer will mean that any initial outlay will be modest. The benefits gained from source data validation for the patients’ existence and consent as well as more stringent blinding to treatment and quality control of the assessment all add value and likely become cost effective in medium- to large-scale trials.

Furthermore, although the approach is still evolving, the use of centralized adjudication begets improvements in inter-rater reliability. Evidence to date suggests that centralized adjudication of the mRS can improve the inter-rater variability in multicenter trials from κ = 0.25 to 0.59 with an ICC = 0.87 for one rater and predicted to be 0.92 with four raters (37). This improvement in reliability can have a modest effect in the reduction of sample size required to see treatment effect. With the high per-patient cost in clinical trials, any potential reduction in patient numbers without sacrificing trial power is of benefit to trialists.

## Conclusion

There are many functional assessment scales available for use in stroke trials. It is possible that previous inappropriate choice of functional outcome assessment may have caused us to miss potential treatment effects in stroke trials. With some thought on the aspect of function of greatest interest (impairment, activity, participation), the preferred psychometric properties, and the proposed analytical technique, the researcher can make an informed choice as to the optimal outcome assessment for their study. The use of novel statistical techniques, rater training, and central adjudication have all been proven to improve the utility of outcomes assessments.

The stroke community has made substantial progress in outcome assessment methodology, but there is still more to do. The outcomes described are poor measures of cognitive and psychological outcomes and yet these are the outcomes of most importance to patients. As we make greater use of “big data,” for example, national registers, we need methods to incorporate feasible but valid outcome assessment into routine data collection.

## Author Contributions

MT-R and TQ drafted the paper. AW and JD contributed to writing, editing, and provided intellectual input.

## Conflict of Interest Statement

TQ and JD assisted in design and validation of training materials for mRS and BI. TQ, JD, and AW have assisted in development, validation, and implementation of video-based mRS assessment.

## Footnotes

^1^https://www.commondataelements.ninds.nih.gov/Doc/NOC/Modified_Rankin_Scale_NOC_Public_Domain.pdf (Accessed: November 30, 2017).

^2^https://secure.trainingcampus.net/uas/modules/trees/windex.aspx?rx=rankin-english.trainingcampus.net (Accessed: November 30, 2017).
