# Supplementary material for: Functional Assessment for Acute Stroke Trials: Properties, Analysis, and Application
Source: Front Neurol. 2018 Mar 26;9:191. doi: 10.3389/fneur.2018.00191 (PMC5879151; doi:10.3389/fneur.2018.00191)
Supplement: Supplementary file 1 [file table_1.docx]

**SUPPLEMENTARY MATERIALS**

Supplementary Figure 1. Barthel Index

| **BARTHEL INDEX** | |
| --- | --- |
| **Activity** | **Score** |
| FEEDING  0 = unable  5 = needs help cutting, spreading butter, etc., or requires modified diet  10 = independent |  |
| BATHING  0 = dependent  5 = independent (or in shower) |  |
| GROOMING  0 = needs to help with personal care  5 = independent face/hair/teeth/shaving (implements provided) |  |
| DRESSING  0 = dependent  5 = needs help but can do about half unaided 10 = independent (including buttons, zips, laces, etc.) |  |
| BOWELS  0 = incontinent (or needs to be given enemas)  5 = occasional accident 10 = continent |  |
| BLADDER  0 = incontinent, or catheterized and unable to manage alone  5 = occasional accident  10 = continent |  |
| TOILET USE  0 = dependent  5 = needs some help, but can do something alone  10 = independent (on and off, dressing, wiping) |  |
| TRANSFERS (BED TO CHAIR AND BACK)  0 = unable, no sitting balance  5 = major help (one or two people, physical), can sit  10 = minor help (verbal or physical)  15 = independent |  |
| MOBILITY (ON LEVEL SURFACES)  0 = immobile or < 50 yards  5 = wheelchair independent, including corners, > 50 yards  10 = walks with help of one person (verbal or physical) > 50 yards  15 = independent (but may use any aid; for example, stick) > 50 yards |  |
| STAIRS  0 = unable  5 = needs help (verbal, physical, carrying aid)  10 = independent |  |
|  | **TOTAL (0–100):** |

Supplementary figure 2. modified Rankin Scale

| **MODIFIED RANKIN SCALE (MRS)**   \| Score Description \| 0 No symptoms at all \| \| --- \| --- \| \| 1 \| No significant disability despite symptoms; able to carry out all usual duties and activities without assistance \| \| 2 \| Slight disability; unable to carry out all previous activities, but able to look after own affairs \| \| 3 \| Moderate disability; requiring some help, but able to walk without assistance \| \| 4 \| Moderately severe disability; unable to walk without assistance and unable to attend to own bodily needs without assistance \| \| 5 \| Severe disability; bedridden, incontinent and requiring constant nursing care and attention \| \| 6 \| Dead \|   TOTAL (0–6): _______ |
| --- | --- | --- | --- | --- | --- | --- | --- | --- | --- | --- | --- | --- | --- | --- |

Supplementary figure 3. NIHSS

| **NIH Stroke Scale**  **Level of Consciousness**  **1a**   \| **Scale Definition** \| \| \| --- \| --- \| \| **0** \| Alert; keenly responsive. \| \| **1** \| Not alert; but arousable by minor stimulation to obey, answer, or respond. \| \| **2** \| Not alert; requires repeated stimulation to attend, or is obtunded and requires strong or painful stimulation to make movements (not stereotyped). \| \| **3** \| Responds only with reflex motor or autonomic effects, or totally unresponsive, flaccid, and areflexic. \|   **1b**   \| **Scale Definition** \| \| \| --- \| --- \| \| **0** \| Answers both questions correctly. \| \| **1** \| Answers one question correctly. \| \| **2** \| Answers neither question correctly. \|   **1c**   \| **Scale Definition** \| \| \| --- \| --- \| \| **0** \| Performs both tasks correctly. \| \| **1** \| Performs one task correctly. \| \| **2** \| Performs neither task correctly. \|   **2**  **Best Gaze**   \| **Scale Definition** \| \| \| --- \| --- \| \| **0** \| Normal. \| \| **1** \| Partial gaze palsy; gaze is abnormal in one or both eyes, but forced deviation or total gaze paresis is not present. \| \| **2** \| Forced deviation, or total gaze paresis is not overcome by the oculocephalic maneuver. \|   **3** **Visual**   \| **Scale Definition** \| \| \| --- \| --- \| \| **0** \| No visual loss. \| \| **1** \| Partial hemianopia. \| \| **2** \| Complete hemianopia. \| \| **3** \| Bilateral hemianopia (blind including cortical blindness). \|   **4** **Facial Palsy**   \| **Scale Definition** \| \| \| --- \| --- \| \| **0** \| Normal symmetrical movements. \| \| **1** \| Minor paralysis (flattened nasolabial fold, asymmetry on smiling). \| \| **2** \| Partial paralysis (total or near-total paralysis of lower face). \| \| **3** \| Complete paralysis of one or both sides (absence of facial movement in the upper and lower face). \|   **5** **Motor Arm**   \| **Scale Definition** \| \| \| --- \| --- \| \| **0** \| No drift; limb holds 90 (or 45) degrees for full 10 seconds. \| \| **1** \| Drift; limb holds 90 (or 45) degrees, but drifts down before full 10 seconds; does not hit bed or other support. \| \| **2** \| Some effort against gravity; limb cannot get to or maintain (if cued) 90 (or 45) degrees, drifts down to bed, but has some effort against gravity. \| \| **3** \| No effort against gravity; limb falls. \| \| **4** \| No movement. \| \| **UN** \| Amputation or joint fusion, explain: \|   **6** **Motor Leg**   \| **Scale Definition** \| \| \| --- \| --- \| \| **0** \| No drift; leg holds 30-degree position for full 5 seconds. \| \| **1** \| Drift; leg falls by the end of the 5- second period but does not hit the bed. \| \| **2** \| Some effort against gravity; leg falls to bed by 5 seconds but has some effort against gravity. \| \| **3** \| No effort against gravity; leg falls to bed immediately. \| \| **4** \| No movement. \| \| **UN** \| Amputation or joint fusion, explain: \|   **7** **Limb Ataxia**   \| **Scale Definition** \| \| \| --- \| --- \| \| **0** \| Absent. \| \| **1** \| Present in one limb. \| \| **2** \| Present in two limbs. \| \| **UN** \| Amputation or joint fusion, explain: \|   **8** **Sensory**   \| **Scale Definition** \| \| \| --- \| --- \| \| **0** \| Normal; no sensory loss. \| \| **1** \| Mild-to-moderate sensory loss; patient feels pinprick is less sharp or is dull on the affected side; or there is a loss of superficial pain with pinprick, but patient is aware of being touched. \| \| **2** \| Severe or total sensory loss; patient is not aware of being touched in the face, arm, and leg. \|   **9** **Best Langauge**   \| **Scale Definition** \| \| \| --- \| --- \| \| **0** \| No aphasia; normal. \| \| **1** \| Mild-to-moderate aphasia; some obvious loss of fluency or facility of comprehension, without significant limitation on ideas expressed or form of expression. Reduction of speech and/or comprehension, however, makes conversation about provided materials difficult or impossible. For example, in conversation about provided materials, examiner can identify picture or naming card content from patient's response. \| \| **2** \| Severe aphasia; all communication is through fragmentary expression; great need for inference, questioning, and guessing by the listener. Range of information that can be exchanged is limited; listener carries burden of communication. Examiner cannot identify materials provided from patient response. \| \| **3** \| Mute, global aphasia; no usable speech or auditory comprehension. \|   **10** **Dysarthria**   \| **Scale Definition** \| \| \| --- \| --- \| \| **0** \| Normal. \| \| **1** \| Mild-to-moderate dysarthria; patient slurs at least some words and, at worst, can be understood with some difficulty. \| \| **2** \| Severe dysarthria; patient's speech is so slurred as to be unintelligible in the absence of or out of proportion to any dysphasia, or is mute/anarthric. \| \| **UN** \| Intubated or other physical barrier, explain: \|   **11** **Extinction and Inattention (formerly Neglect)**   \| **Scale Definition** \| \| \| --- \| --- \| \| **0** \| No abnormality. \| \| **1** \| Visual, tactile, auditory, spatial, or personal inattention, or extinction to bilateral simultaneous stimulation in one of the sensory modalities. \| \| **2** \| Profound hemi-inattention or extinction to more than one modality; does not recognize own hand or orients to only one side of space. \| |
| --- | --- | --- | --- | --- | --- | --- | --- | --- | --- | --- | --- | --- | --- | --- | --- | --- | --- | --- | --- | --- | --- | --- | --- | --- | --- | --- | --- | --- | --- | --- | --- | --- | --- | --- | --- | --- | --- | --- | --- | --- | --- | --- | --- | --- | --- | --- | --- | --- | --- | --- | --- | --- | --- | --- | --- | --- | --- | --- | --- | --- | --- | --- | --- | --- | --- | --- | --- | --- | --- | --- | --- | --- | --- | --- | --- | --- | --- | --- | --- | --- | --- | --- | --- | --- | --- | --- | --- | --- | --- | --- | --- | --- | --- | --- | --- | --- | --- | --- | --- | --- | --- | --- | --- | --- | --- | --- | --- | --- | --- | --- | --- | --- | --- | --- | --- | --- | --- | --- | --- | --- | --- | --- | --- | --- | --- | --- | --- | --- |
